# Obesity in the Otsuka Long Evans Tokushima Fatty Rat: Mechanisms and Discoveries

**DOI:** 10.3389/fnut.2016.00021

**Published:** 2016-07-27

**Authors:** Sheng Bi, Timothy H. Moran

**Affiliations:** ^1^Department of Psychiatry and Behavioral Sciences, Johns Hopkins University School of Medicine, Baltimore, MD, USA; ^2^Global Obesity Prevention Center, Johns Hopkins Bloomberg School of Public Health, Baltimore, MD, USA

**Keywords:** cholecystokinin, neuropeptide Y, CCK-1 receptor, dorsomedial hypothalamic nucleus, food intake, obesity

## Abstract

Understanding the neural systems underlying the controls of energy balance has been greatly advanced by identifying the deficits and underlying mechanisms in rodent obesity models. The current review focuses on the Otsuka Long Evans Tokushima Fatty (OLETF) rat obesity model. Since its recognition in the 1990s, significant progress has been made in identifying the causes and consequences of obesity in this model. Fundamental is a deficit in the cholecystokinin (CCK)-1 receptor gene resulting in the absence of CCK-1 receptors in both the gastrointestinal track and the brain. OLETF rats have a deficit in their ability to limit the size of meals and in contrast to CCK-1 receptor knockout mice, do not compensate for this increase in the size of their spontaneous meals, resulting in hyperphagia. Prior to becoming obese and in response to pair feeding, OLETF rats have increased expression of neuropeptide Y (NPY) in the compact region of the dorsomedial hypothalamus (DMH), and this overexpression contributes to their overall hyperphagia. Study of the OLETF rats has revealed important differences in the organization of the DMH in rats and mice and elucidated previously unappreciated roles for DMH NPY in energy balance and glucose homeostasis.

## Introduction

Rodent obesity models have been critical to our understanding of the neural systems involved in the controls of food intake and body weight. Dissection of the genetics underlying the obesity of ob/ob and db/db mice led not only to the discovery of leptin but also contributed greatly to the understanding of multiple hypothalamic peptide systems involved in energy balance. Another example of a genetic model that has increased our understanding of the neural systems involved in energy balance is the Otsuka Long Evans Tokushima Fatty (OLETF) rat. This rat obesity model was derived from a spontaneous obesity in an outbred colony of Long Evans rats. OLETF and a control Long Evans Tokushima Otsuka (LETO) lines were then developed by selective breeding. OLETF rats were initially studied primarily as a model of late onset type 2 diabetes, as older OLETF rats were not only obese but also hyperglycemic and insulin resistant (1).

Characterization of overall pancreatic function in OLETF rats demonstrated the absence of a pancreatic amylase response to administration of the brain gut peptide cholecystokinin (CCK) (2). Further studies revealed that OLETF rats had a >6 kbp deletion in the gene for the CCK-1 receptor that spanned the first and second exons and resulted in the absence of expression of a functional CCK-1 receptor (3). Thus, the OLETF rat is a CCK-1 receptor knockout model.

## Cholecystokinin and Cholecystokinin Receptors

Cholecystokinin is a gut/brain peptide that plays a variety of roles. Gut CCK is released from I cells in the upper intestine in response to the intraluminal presence of nutrients and plays a variety of roles in the overall digestive function. Exogenously administered and endogenously released CCK slow gastric emptying, modulate intestinal motility and stimulate gall bladder and pancreatic secretions. CCK also plays a role in the control of food intake by contributing to meal termination. Exogenously administered CCK reduces food intake and does so by reducing meal size (4–6). A role for endogenously released CCK in the controls of meal size is demonstrated by the ability of CCK receptor antagonists to increase food intake by prolonging eating – increasing meal duration and size (7, 8). The primary mechanism of action of CCK in the inhibition of food intake is paracrine, acting on local vagal afferent terminals in close apposition to the intestinal I cells (9, 10). CCK receptors are expressed in vagal afferent cell bodies in the nodose ganglion and transported to abdominal vagal endings (11). CCK both directly activates vagal afferent fibers and also sensitizes vagal fibers to signals, transmitting information about gastric and intestinal luminal volume (12, 13).

In the brain, CCK acts as neurotransmitter/neuromodulator. CCK-producing neurons are widely distributed in the brain, and CCK neurons have been reported to be the most ubiquitous of all peptidergic neurons. Cell bodies are found throughout all layers of the cerebral cortex and are widely distributed throughout olfactory and limbic systems and in multiple hypothalamic nuclei. In the midbrain, CCK cell bodies are found in the substantia nigra, the ventral tegmental area, and the raphe nucleus (14, 15), and CCK modulates both dopaminergic and serotonergic function (16).

There are two CCK receptor subtypes (17, 18). These were initially identified based on their relative affinity for various CCK fragments and analogs. CCK-1 receptors require the sulfated tyrosine, and these were originally characterized in rat and guinea pig pancreas. CCK-1 receptors exist in both low capacity, high affinity and high capacity, low affinity states. CCK-2 receptors have high affinity for unsulfated CCK and various CCK fragments and were initially characterized in brain. Both receptors are members of the G-coupled super family of receptors. As well as found in pancreas and gall bladder, CCK-1 receptors are expressed in the nodose ganglion (and transported in vagal afferent fibers) and in a number of specific brain sites, including the dorsomedial hypothalamus (DMH) (17). There are important species-specific differences in the expression patterns of CCK-1 and CCK-2 receptors, including the expression of CCK-2 and not CCK-1 receptors in human pancreas. However, the expression of CCK-1 receptors in vagal afferent neurons and in specific brain sites appears to be similar in rat and man (not in the mouse as will be discussed later).

The satiety actions of CCK depend on the interactions with CCK-1 receptors. Sulfated CCK-8 or sulfated longer forms (i.e., CCK-33, CCK-58) inhibit food intake in a dose-related fashion, while unsulfated CCK or shorter CCK fragments do not (4, 19). Furthermore, specific CCK-1 antagonist administration increases food intake while CCK-2 antagonists do not (7). This pharmacological specificity has been demonstrated across multiple species.

## Characterization of the Hyperphagia in OLETF Rats

**Figure 1:**
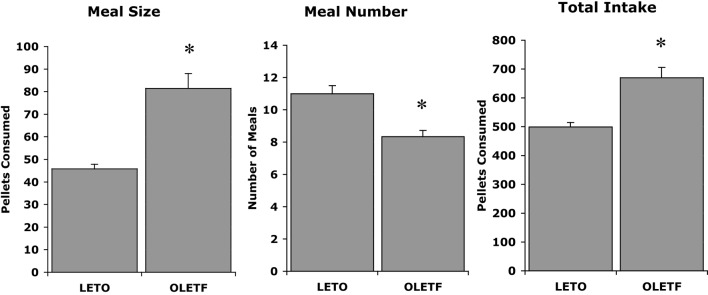
**Meal patterns in LETO and OLETF rats (20)**.

The initial discovery that OLETF rats had a deletion in the gene for the CCK-1 receptor led to experiments examining whether CCK could inhibit their food intake. OLETF rats lacking functional CCK-1 receptors were shown to be insensitive to the feeding inhibitory actions of exogenously administered CCK. Characterization of their daily food intake revealed that OLETF rats ate meals that were about twice as large as those of LETO controls and, in response to this increase in the size of their meals, they at fewer meals. However, the decrease in meal frequency was not sufficient to normalize their food intake resulting in a chronic hyperphagia or overconsumption (Figure 1) (20). Evidence for the hyperphagia is evident even prior to weaning. In independent ingestion tests, in which rat pups are consuming milk off the floor of a test chamber, OLETF pups as young as 2 days of age consume significantly more sweetened milk than age-matched LETO controls (21). In tests assessing nursing behavior, OLETF pups also gain more weight during a suckling bout indicative of increased intake (22).

The food intake of OLETF rats is also characterized by higher preferences for high fat (23), sucrose and other sweet tastes (24). This can be demonstrated in both real feeding and sham feeding paradigms, implicating taste mechanisms in the preferences.

**Figure 2:**
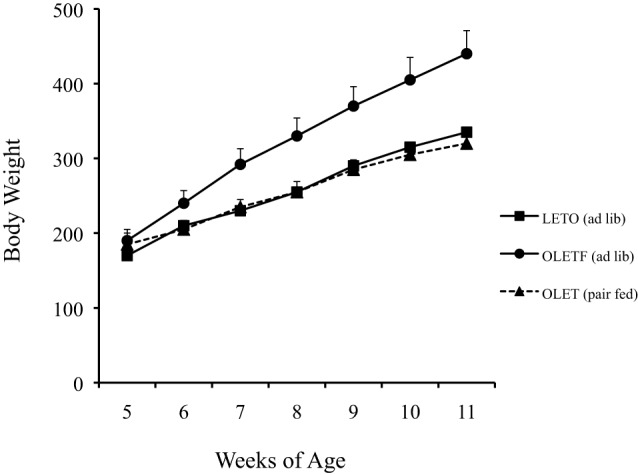
**Pair feeding normalized body weight in growing OLETF rats (20)**.

Pair feeding experiments in which the daily intake of OLETF rats was limited to that of paired LETO control rats revealed that the obesity in the OLETF rats was completely attributable to their hyperphagia. Pair feeding completely normalized their rates of body weight gain (Figure 2) as well as the size of their fat mass and their glucose regulation (20). Thus, the OLETF rat is an obesity model of disordered food intake.

## Characterization of Hypothalamic Function in OLETF Rats

The lack of compensation for the increase in meal size in OLETF rats requires explanation. Chronic administration of CCK at meal onset results in chronic decreases in meal size but an increase in meal frequency such that overall food intake is not affected (5). These data suggest a role for CCK in meal termination, but not in overall food intake. Knockout of CCK-1 receptors in the mouse produces results that are consistent with this interpretation. CCK-1 knockout mice have increased meal size, but the decrease in meal frequency compensates for this so that CCK-1 KO mice have normal body weight (25, 26). Why does the absence of CCK-1 receptors result in obesity in the OLETF rat, but not in a mouse KO?

Part of the answer comes from the examination of hypothalamic signaling in the OLETF rat. While mRNA expression for arcuate POMC and neuropeptide Y (NPY) was appropriate in obese or lean pair-fed OLETF rats [elevated POMC and reduced NPY in the obese state and normal expression in lean OLETF rats pair-fed to amounts consumed by control LETO rats (27)], NPY expression in the compact subregion of the DMH was significantly elevated in pair-fed OLETF rats and normalized in ad lib-fed rats (27). These data suggested the possibility that elevations in DMH NPY might be driving the hyperphagia on OLETF rats. Analyses of NPY expression levels in juvenile OLETF rats prior to obesity were consistent with such an explanation. Five-week-old pre-obese OLETF rats had greatly elevated DMH NPY expression. Importantly, the same neurons expressing NPY in the DMH also expressed CCK-1 receptors representing one of the populations of brain CCK-1 receptors identified in the original autoradiography studies (27). Furthermore, direct injection of CCK into the DMH both reduces food intake and downregulates NPY mRNA expression without affecting ARC NPY expression, suggesting a role for CCK in modulating DMH NPY (28). In the absence of CCK-1 receptors, DMH NPY is upregulated.

An examination of NPY expression in the mouse revealed that although NPY expression was evident in the ARC, its expression was not evident in the compact region of the DMH. NPY receptors are evident in the dorsal and ventral medial subregions of the DMH, and NPY expression increases in response to exposure to a high-fat diet. A role for these in the lasting hyperphagia that occurs in diet-induced obesity has been suggested (29). In contrast to rats, mouse DMH does not contain CCK-1 receptors as neither binding activity nor mRNA expression, for CCK-1 receptors are detected in the DMH (23). These data have led to the hypothesis that the obesity in the OLETF rats results from a combination of disordered satiety signaling due to the lack of vagal afferent CCK-1 receptors and an upregulation of DMH NPY that prevents complete compensation for the increased meal size. The CCK-1 receptor knockout mouse has similar deficits in the control of meal size, but in the absence of altered DMH signaling, appropriately compensates for chronically consuming larger meals.

This hypothesis was directly tested in the rat using viral-mediated knockdown of DHM NPY in OLETF rats (30). Forty percent knockdown of DMH NPY mRNA expression in response to bilateral administration of an AAV-expressing short hairpin RNA (AAVshNPY) significantly reduced the food intake and weight gain trajectory of OLETF rats. The alteration in food intake was expressed as a partial reduction in the size of consumed meals such that the meal size deficit in OLETF rats with DMH injections of AAVshNPY was similar to the meal size deficits in CCK-1 receptor KO mice. DMH NPY overexpression in control rats had the opposite effect. Overexpressing DMH NPY resulted in increased food intake, especially on a high-fat diet, and significantly elevated weight gain (30).

## Exercise and OLETF Obesity

The study of the OLETF rat has led to a number of important insights about interactions between exercise and food intake and the role of DMH signaling in energy balance. Providing OLETF rats access to a running results in a normalization of their body weight (31) and prevention if hyperinsulinemia (32). This is not simply due to the increased energy expenditure as their daily food intake is also greatly reduced by running wheel access, and their meal patterns are normalized (33). The long-term effects of running wheel activity depend upon the timing of access. In adult OLETF rats, running wheel access normalizes food intake and body weight, but at the cessation of access, food intake greatly increases, and body weight returns to levels of comparably aged OLETF rats that did not have access to running wheels. Thus, the effects of exercise are temporary and only evident during the time of running wheel access. In contrast, providing access to running wheels for a 6-week period beginning at 8 weeks of age had long-lasting effects on both food intake and body weight in OLETF rats. Although food intake and body weight increased somewhat when access to the running wheels was stopped, OLETF rats did not regain weight to levels of control OLETF rats without running wheel access (33). Effects of exercise on other rodent obesity phenotypes have now been demonstrated as well (34–36). The age-dependent aspect of the effects of exercise may depend on epigenetic effects in pathways undergoing maturation and thus increasing the possibility of lasting effects when the exposure is at a younger age.

## Novel Actions of DMH NPY

The observation of altered DMH NPY signaling in the OLETF rat and how DMH knockdown rescues the obese phenotype has led to extensive studies of the roles of DMH NPY in various aspects of energy balance. As mentioned above, overexpression of DMH NPY leads to increased food intake and body weight, especially when rats are presented with a high-fat diet (30).

These data led to a more careful examination of the consequences of altered DMH NPY signaling. Knockdown of NPY in the DMH in normal weight Sprague-Dawley rats has been demonstrated to reduce the size of fat depots and ameliorate high-fat diet-induced hyperphagia and obesity. Furthermore, DMH NPY knockdown resulted in the development of brown adipocytes in inguinal white adipose tissue that was characterized by increased uncoupling protein 1 expression. DMH NPY knockdown also increased energy expenditure and enhanced the thermogenic response to a cold environment. This knockdown also enhanced insulin sensitivity. These data identified novel roles for DMH NPY in modulating adipose tissue, thermogenesis, insulin sensitivity, and energy expenditure (37).

Further work has revealed a novel modulator of DMH NPY signaling. Gene expression profiling of the DMH in response to exercise revealed an elevation of the expression of transthyretin (TTR), best known as a blood and cerebrospinal fluid transporter of thyroxine and retinol. To test the hypothesis that TTR may play a role in modulating signaling-related energy balance in the DMH, we examined the effects of brain TTR on food intake and body weight and have further determined hypothalamic signaling that may underlie its feeding effect in rats. We found that icv administration of TTR in normal growing rats decreased food intake and body weight. Furthermore, TTR administration decreased NPY levels in the DMH. Chronic icv infusion of TTR in OLETF rats reversed their hyperphagia and obesity. Overall, these studies examining factors that might modulate DMH NPY demonstrated a novel anorectic action of central TTR in the control of energy balance (38), providing a potential novel target for obesity treatment.

## Summary

Work with the OLETF rat has not only been focused on identifying the mechanisms underlying its obesity but also served as a vehicle for uncovering multiple novel mechanisms involved in the overall controls of energy balance.

## Author Contributions

Both authors have approved the final version of the manuscript.

## Conflict of Interest Statement

The authors declare that the research was conducted in the absence of any commercial or financial relationships that could be construed as a potential conflict of interest.
